# Isolation and primary culture of *Galleria mellonella *hemocytes for infection studies

**DOI:** 10.12688/f1000research.27504.2

**Published:** 2021-02-16

**Authors:** Nicola J. Senior, Richard W. Titball

**Affiliations:** 1 College of Life and Environmental Sciences - Biosciences, University of Exeter, Exeter, Devon, EX4 4QD, UK

**Keywords:** Galleria mellonella, hemocytes, 3Rs, infection model

## Abstract

*Galleria mellonella* larvae are increasingly used to study the mechanisms of virulence of microbial pathogens and to assess the efficacy of antimicrobials.  The *G. mellonella* model can faithfully reproduce many aspects of microbial disease which are seen in mammals, and therefore allows a reduction in the use of mammals. The model is now being widely used by researchers in universities, research institutes and industry. An attraction of the model is the interaction between pathogen and host. Hemocytes are specialised phagocytic cells which resemble neutrophils in mammals and play a major role in the response of the larvae to infection. However, the detailed interactions of hemocytes with pathogens is poorly understood, and is complicated by the presence of different sub-populations of cells. We report here a method for the isolation of hemocytes from *Galleria mellonella*.  A needle-stick injury of larvae, before harvesting, markedly increased the recovery of hemocytes in the hemolymph. The majority of the hemocytes recovered were granulocyte-like cells. The hemocytes survived for at least 7 days in culture at either 28°C or 37°C. Pre-treatment of larvae with antibiotics did not enhance the survival of the cultured hemocytes. Our studies highlight the importance of including sham injected, rather than un-injected, controls when the *G. mellonella* model is used to test antimicrobial compounds. Our method will now allow investigations of the interactions of microbial pathogens with insect hemocytes enhancing the value of *G. mellonella* as an alternative model to replace the use of mammals, and for studies on hemocyte biology.

Research highlights **Scientific benefits:**

- Allows the interactions of microbial pathogens with host insect hemocytes to be investigated ex vivo.
- Allows the biology of different types of hemocytes to be investigated ex vivo.
- Allows comparative studies of the interaction of hemocytes and mammalian phagocytes with pathogens.

**3Rs benefits:**

- *G. mellonella* larvae are increasingly used as alternatives to mice and other mammals for studies on the mechanisms by which microbial pathogens cause disease and to test pre-treatments and therapies for disease.

**Practical benefits:**

- The protocol described allows the isolation of higher yields of hemocytes from *G. mellonella* for experimental studies using a simple technique.
- The protocol described establishes the optimum time window for using isolated hemocytes in experimental studies.

**Current applications:**

- Infectious disease research to understand mechanism of virulence of microbial pathogens.

**Potential applications:**

- Testing the ability of isolated hemocytes to support the growth of intracellular pathogens including *Burkholderia* sp, *Coxiella burnetii*, *Mycobacterium* sp. *Salmonella enterica*.
- Using the hemocyte model to assess the ability of drugs to target intracellular pathogens.
- Using the hemocyte model to compare the virulence of different isolates and different mutants of microbial pathogens.

## Introduction

Improving our understanding of infectious disease, developing new pre-treatments and therapies for diseases and testing the safety of biological and chemical materials often requires the use of regulated vertebrate animals. Typically, mammals are used for these studies. There is a need to replace, refine and reduce the use of regulated animal species in the UK and one approach is to develop alternative test systems and models for infectious disease.

A wide range of alternatives have been proposed and developed, and these have different benefits and drawbacks ( Table 1). Larvae of the greater waxmoth *Galleria mellonella* are an attractive alternative because they can be injected with precise doses of pathogen or chemical compound, and incubated at 37°C to mimic conditions in a mammalian host ^1^. The model is becoming well developed and the late-stage larvae, which are used for research and testing, do not require food or water and are easy to maintain. Compared to regulated animals, the larvae are ethically more acceptable and more cost effective ^2^, and their use can contribute to the target to reduce and replace the use of regulated animals. *G. mellonella* have been used to study virulence of pathogens, as part of the drug discovery pipeline and in chemical and drug toxicity testing ^1– 5^. The model is now used widely in academia and increasingly used by pharmaceutical industries drug and contract research organisations. Although *G. mellonella* larvae can never replace mammalian models completely, the rise in usage over the past decade, with 275 publications using *G. mellonella* in 2019, indicates how valuable the larvae have become to researchers. This work reported here is part of a project to develop the *G. mellonella* model to reduce the number of mammals used in research associated with insect-vectored viral pathogens. Regulated vertebrate models are frequently used to study viral disease, and to develop and evaluate therapeutics. To assess the use of mammals for virus research in the UK we carried out a PubMed search using the key words “virus+UK+mice”. This returned 118 relevant publications in 2018. We selected the first five publications for which we could obtain journal access at the University of Exeter, and found that the total number of mice used in these five publications was 235. In addition eight rabbits and six marmosets had been used in these studies. Assuming that these numbers are representative for all publications in 2018, we calculate that in the UK at least 5546 mice were used for studies in viral diseases in 2018. In addition at least 189 rabbits and 141 non-human primates were used in 2018. The procedures are judged to be severe. For example, in one laboratory almost 1000 mice were used to study viral pathogenesis and to evaluate therapies. We envisage that up to 50% of these animals could be replaced by *G. mellonella* larvae if a suitable infection model was available. Although studies carried out in *G. mellonella* larvae will never completely replace studies in mammals, they will allow any subsequent work using regulated mammals to be better designed, providing refinement of experiments.

**Table 1.** Living systems which have been used as experimental alternatives to vertebrate animals.

Model	Whole animal model	Model well developed?	Use at 37°C?	Precise dosing?	Cost of maintenance by user	Regulated in the UK?
Monolayer cell cultures	No	Yes	Yes	Yes	Medium	No
3D cell cultures	No	Limited	Yes	Yes	High	No
*Caenorhabditis* *elegans*	Yes	Yes	No	No	Low	No
*Panagrellus redivivus*	Yes	No	Limited	No	Low	No
*Danio rerio* embryos	Yes	Limited	No	Yes	High	Not for early stage embryos
*Drosophila* *melanogaster*	Yes	Limited	Yes	No	Low	No
*Galleria mellonella*	Yes	Yes	Yes	Yes	Low	No
*Manduca sexta*	Yes	Yes	Yes	Yes	Low	Regulated by as a crop pest
*Arabidopsis thaliana*	No	No	No	No	Low	No
*Allium cepa*	No	No	No	No	Low	No

One of the advantages of *G. mellonella* is that they provide a whole animal model, rather than being cell culture-based. Additionally, *G. mellonella* possess an innate immune system, involving cellular and humoral responses ^6– 8^. The cellular response involves hemocytes that can engulf pathogens, and these cells share a high degree of structural and functional similarity with mammalian neutrophils ^9^. The humoral response involves activation of Toll and Imd pathways, resulting in the production of antimicrobial peptides ^10^, the production of prophenoloxidase and the generation of melanin ^10^.

The similarities between insect hemocytes and mammalian neutrophils are well-documented ^8– 10^. Like neutrophils, hemocytes use a respiratory burst to generate reactive oxygen species and kill pathogens ^11^. This respiratory burst is triggered by the translocation of p47 ^phox^ and p67 ^phox^ proteins in neutrophils, and by the translocation of 47 and 67 kDa proteins in hemocytes ^11^. Like neutrophils, hemocytes possess Toll-like receptors, β-glucan and interleukin-1 receptors ^11^ and signalling occurs via NFκB pathways ^11^. The antimicrobial peptides produced by neutrophils and hemocytes, in response to signalling, are similar ^11^ and include lysozyme, transferrin and defensins. Finally, hemocytes and neutrophils can produce extracellular traps (NETs) to immobilize and kill pathogens ^12, 13^.

In *G. mellonella* four types of hemocytes have previously been identified: plasmatocytes, granulocytes, spherulocytes and oenocytoids ^14^. A fifth type, prohemocytes ^15^ may be stem cells that differentiate into other hemocyte types ^16^ In larval stage Lepidoptera, granulocytes and plasmatocytes are considered to be the main phagocytic hemocytes, and they are also the most numerous cells in circulation ^9^.

Pathogens that have intracellular lifestyles in mammalian hosts, such as *Coxiella burnetii*, *Burkholderia thailandensis*, *Francisella tularensis* and *Mycobacterium tuberculosis*, have previously been shown to invade hemocytes and cause death in a dose-dependent manner ^17– 19^. However, the interactions of pathogens with hemocytes are poorly understood. This information is required to allow an understanding of the similarities and differences in the ways in which pathogens interact with hemocytes from insects or phagocytes in mammals, and therefore to provide an understanding of the utility of the *G. mellonella* infection model. Here we report the development of a protocol for the isolation and culture of hemocytes from *G. mellonella* to allow the interactions of pathogens with hemocytes to be investigated. The method we have developed will be of interest to researchers using *G. mellonella* as a model to study infectious disease.

## Methods

### Methods for the model development

***Experimental design.*** The experimental unit in this study was a single *Galleria mellonella* larva. The number of larvae per group was estimated using a Resource Equation method ^20^; the assignation of 10 larvae per group across four groups gave an E value of 36, which is greater than the recommended figure of 10–20. Since the use of larvae does not breach ethical guidelines, this could be justified as we had no previous experience regarding the number of hemocytes that could be obtained from a single larva. Treatment was randomised: all treatments were started at the same time; the larvae were selected from a pool of healthy individuals within the weight parameters of 0.18–0.35 g; they were assigned to treatment groups by taking larvae from the pool with no visual reference to compare them to other larvae. All experimental groups were equal in size. There was no blinding of samples for hemocyte counts.

***G. mellonella larvae.*** TruLarv™ final (6 ^th^) instar larvae were purchased from ** BioSystems Technology, Exeter, UK. TruLarv are reared without antibiotics or hormones which are normally added to feedstuffs, and are 0.18 g–0.35 g final instar stage larvae. The larvae were stored at 15°C for up to one week before using in the experiments reported. During storage the larvae do not require food or water.

**Figure 1.**
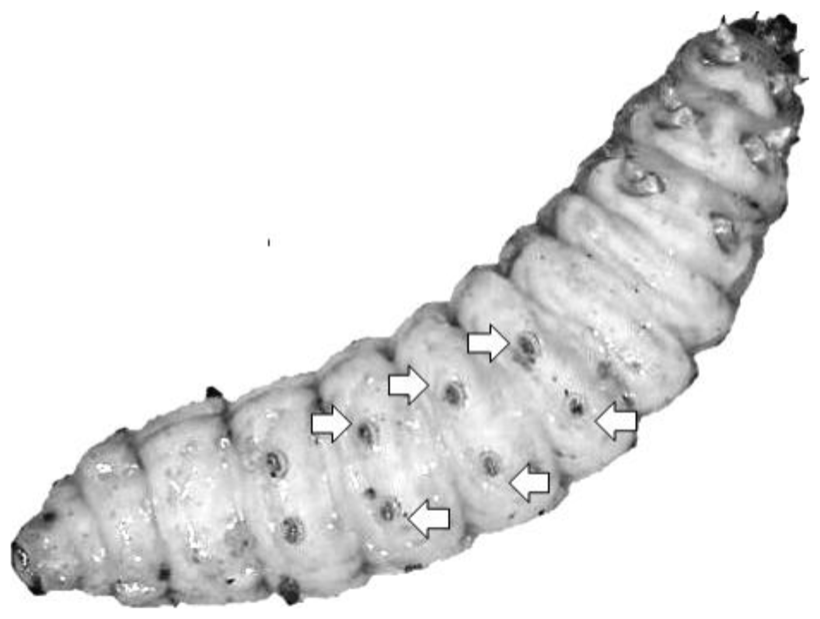
View of a single *G. mellonella* larva from the underside, showing the six frontal prolegs (arrowed) which are the preferred sites for injection.

***Pre-treatment of larvae.*** Groups of ten larvae (TruLarv™) were used in these studies to allow the collection of sufficient hemolymph for the studies outlined below. Typically, we recovered 20–30 µl of hemolymph from each larva. We tested whether pre-dosing with antibiotics would minimise the bacterial flora on the larvae and therefore minimise the possibility of bacterial infection of the isolated hemocyte cultures. Where indicated, the larvae were treated with a scaled human dose of the broad-spectrum antibiotic doxycycline or ciprofloxacin prior to the extraction of the hemolymph. Larvae were swabbed at the injection site with 70% ethanol and the antibiotic (10 μl) injected 2–3mm deep into a proleg ( Figure 1). In some studies the larvae were wounded by piercing the proleg with a 21- or 22-gauge needle. These treatments were repeated daily for up to 7 days, using a different proleg at each time. Larvae were stored in Petri dishes lined with filter paper and kept in the dark at 15°C for the duration of the pre-treatment.

***Hemolymph extraction.*** The main cell population in insect hemolymph is circulating hemocytes, and few other cell types are present. Before hemolymph extraction, larvae were swabbed with 70% ethanol, then positioned over a 1 ml pipette tip. A proleg was pierced 2–3 mm deep with a 21- or 22-gauge needle, and the hemolymph that emerged was collected using a pipettor set to 50 μl. Typically, the concentration of hemocytes in the pooled hemolymph was 2×10 ^6^ to 4×10 ^6^ cells per ml. Since we typically harvested 200–300 µl of hemolymph from 10 larvae, in total we isolated 4×10 ^5^ to 1.6×10 ^6^ cells. The hemolymph was transferred into a microcentrifuge tube containing 500 μl of insect physiological saline (IPS; 150 mM NaCl, 5 mM KCl, 100 mM Tris/HCl, 10 mM EDTA, 30 mM sodium citrate, pH 6.9) on ice. The hemolymph from each group of larvae was pooled and centrifuged at 500 × g, 4°C, for 5 minutes. Pellets were washed twice in ice-cold IPS, then re-suspended in 1 ml ice-cold IPS. A total of 10 µl were withdrawn from suspended pellets and mixed with 10 μl of trypan blue to enable cell enumeration in a hemocytometer. The concentration of hemocytes was adjusted as required and the cells used immediately.

***Maintenance of hemocytes.*** The required amount of cell suspension (at 2 × 10 ^5^ cells/ml) was placed into the relevant number of wells in a 24-well plate, and made up to 1 ml with Grace’s Insect Medium supplemented with L-glutamine and sodium bicarbonate + 2% penicillin-streptomycin + 2.5 μg/ml amphotericin B + 10% heat-inactivated foetal bovine serum (FBS) at room temperature. The plates were placed in a lidded vented box lined with damp blue roll to reduce evaporation and incubated at 28°C under normal atmospheric conditions. Overall, 50% of the medium was replaced every 3–5 days. The plates were observed under 10 × magnification on a microscope regularly for evidence that the cells were intact and remained attached to the bottom of the wells.

### Methods for the characterisation of the *ex vivo* hemocyte model

***Hemocyte quantification.*** Hemocytes were quantified after dissociating them from the wells as detailed below. The medium was removed and the cells washed twice in Dulbecco’s PBS. Next, 200 μl of trypsin + 0.25% EDTA, pre-warmed to 37°C, was added to each well; the plate was incubated at 37°C for 3 minutes. The trypsin was inactivated by the addition of 400 μl of the culture medium, which was pipetted slowly over the well surface at least twice. The base of each well was tapped sharply. If observations under the microscope suggested that the cells still had not dissociated, the base of the well was gently scraped with a 10 μl sterile disposable plastic inoculation loop to release any cells attached to the well bottom. Ten microliters were withdrawn from the cell suspension and mixed with 10 μl of trypan blue to enable viable cell enumeration in a hemocytometer.

***Differential hemocyte counts.*** Hemocytes were examined to ascertain the proportion of different cell types (granulocytes, spherulocytes, plasmatocytes, prohemocytes or oenocytoids). They were collected from larvae injected with doxycycline or sham injected by inserting a needle into a proleg 3 days before harvesting hemolymph. The hemolymph was pooled from 10 larvae per treatment, and aliquots dispensed into three wells on each of two plates. One set of plates was observed for the presence of intact and attached cells under 20 × magnification with the cells unstained at T0. The other set of plates was incubated at 28°C for seven days and then observed under the same conditions.

Hemocytes were also observed by staining *in situ*. Medium was removed from wells and the cells were washed with Dulbecco’s PBS. They were briefly allowed to air-dry and 200 μl of Giemsa Stain was added to the wells to visualise the hemocytes and left for 2 minutes before being removed and replaced with 200 μl of deionized water for 3 minutes. Giemsa stain is a widely used histological stains, colouring nuclei dark blue and the cytoplasm blue or pink depending on the acidity of the cytoplasm. The wells were rinsed with deionized water; as much liquid as possible was removed using a pipette, and the wells were left to air-dry. The wells were observed under 20 × magnification on a microscope and images taken using a camera.

***Statistical analysis of data.*** Statistical analysis was carried out using the GraphPad Prism 8 program (GraphPad Software, LA Jolla California USA). The means and standard deviations were calculated for each set of results. 

### Protocol for the use of this model

Here we describe the step by step procedure used to extract hemocytes and their use as an *ex vivo* model. Reagents are listed in Table 2.

**Table 2.** Reagents and small laboratory equipment used in this protocol.

Reagent	Supplier	Supplier #
Doxycycline hyclate	Sigma Aldrich	D9891-5G
Ciprofloxacin	Sigma Aldrich	17850-5G-F
Hydrochloric acid	Fisher Scientific	11393777
Sodium chloride	Fisher Scientific	10553515
Potassium chloride	Sigma Aldrich	P3911-500G
Tris	Fisher Scientific	10376743
EDTA	Fisher Scientific	10203570
Sodium citrate	Sigma Aldrich	S4641-25G
Trypan blue	Sigma Aldrich	T8154-20ML
Grace’s insect medium supplemented with L-glutamine and sodium bicarbonate	Sigma Aldrich	G8142-500ML
Penicillin-streptomycin solution	Sigma Aldrich	P4333-20ML
amphotericin B solution	Sigma Aldrich	A2942-20ML
Foetal bovine serum	Pan Biotech	P40-37500HI
Trypsin + 0.25% EDTA	Fisher Scientific	10693313
Dulbecco’s phosphate-buffered saline (PBS)	Sigma Aldrich	D8537-500ML
Giemsa stain	Sigma Aldrich	48900-100ML-F
Small laboratory equipment	Supplier	Supplier #
22-gauge cemented-needle Hamilton syringe	Cole-Palmer	WZ-07939-01
21-gauge sterile syringe needles	Becton Dickinson	305145
24-well cell culture plates (Corning™ Costar™ Flat Bottom Cell Culture Plates)	Fisher Scientific	10380932
Hemocytometer	Sigma Aldrich	Z359629-1EA
Hemocytometer cover slips	Sigma Aldrich	Z375357-1EA

****Step 1: Preparation of antibiotic stocks (Day 1)****. We use doxycycline hyclate to make up a stock solution of doxycycline (8.5 mg in 1 ml) of sterile milliQ water. This is diluted to 60 µg in 1 ml of sterile milliQ water to ensure a scaled human dose (on a weight for weight basis) will be given to larvae (equivalent to a dose of 0.6 µg in 10 µl), × 3. We use ciprofloxacin to prepare a stock solution (10 mg in 1 ml of 0.1N hydrochloric acid). This is diluted to 250 µg in 1 ml of sterile milliQ water (equivalent to a dose of 2.5 µg in 10 µl), × 3. The stock solutions are stored at -20°C and scaled human doses are used on the day of dilution and also frozen in aliquots.

**Figure 2.**
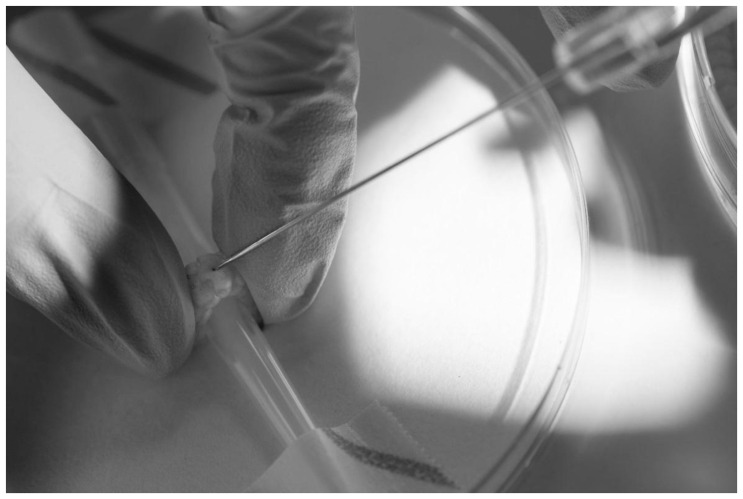
Technique for immobilisation of larvae for the injection or withdrawal of fluids.

****Step 2: Pre-treatment of larvae (Day 1)****. We use TruLarv™ (BioSystems Technology, Exeter, UK), weight 0.18–0.35 g, 10 larvae per treatment. These are maintained in an incubator at 15°C before and during treatment. An infection station is set up comprising a 90 mm filter paper taped to the bench with a 1-ml pipette tip taped across it ( Figure 2). Larva are held over the pipette tip, underside uppermost, and swabbed with 70% ethanol. Next, 10 µl of the doxycycline dilution is drawn into a 22-gauge cemented-needle Hamilton syringe; this is injected into one of the larval prolegs. The syringe is lifted with the larvae still attached and held over a Petri dish lid lined with a 90-mm filter paper; the larva is allowed to free itself into the lid. This is repeated for the other nine larvae, and the base of the petri dish used as the lid. The ciprofloxacin solution is injected into another 10 larvae. Another 10 larvae are wounded by piercing a proleg with the syringe needle, but without corresponding injection, and these are held in a third Petri dish. A further 10 larvae are put into a fourth Petri dish as the untreated controls. All dishes are then stored in the incubator at 15°C for 24 hours.

****Step 3: Pre-treatment of larvae (Day 2)****. Aliquots of diluted antibiotic are removed from -20°C storage and thawed. The larvae from the previous day are removed from the incubator, and an infection station is prepared as detailed above. The larvae are swabbed with 70% ethanol before their respective treatments, which are injected into a different proleg to that used on the previous day. The larvae are returned to the 15°C incubator for 24 hours.

****Step 4: Pre-treatment of larvae (Day 3)****. An aliquot of diluted antibiotic is removed from -20°C and thawed. The larvae in dishes are removed from the incubator, and an infection station is prepared. The larvae are again swabbed with 70% ethanol prior to their respective treatments, which are injected into a third proleg. The larvae are returned to the 15°C incubator for at least 1 hour.

****Step 5: Extraction of hemolymph (Day 3)****. The larvae are removed from the 15°C incubator. Next, 500 µl of insect physiological saline (IPS; 150 mM NaCl, 5 mM KCl, 100 mM Tris/HCl, 10 mM EDTA, 30 mM sodium citrate, pH 6.9) is added into each of four microcentrifuge tubes that are placed on ice. A 1-ml pipette tip is placed into the lid of a Petri dish. In turn, each larva from the doxycycline set is swabbed with 70% ethanol and held backwards over the pipette tip. A different proleg is pierced with a 21-gauge needle, and the hemolymph that emerges is drawn into a pipette tip on a 200 µl pipettor set to 50 µl. The hemolymph is pooled into the microcentrifuge tube containing ice-cold IPS. In the same way, the hemolymph extracted from the ciprofloxacin, wounded and untreated sets are pooled into separate microcentrifuge tubes on ice. The pools are centrifuged at 500 × g, 4°C, for 5 minutes, then supernatants are discarded. Pellets are washed in 1 ml ice-cold IPS and re-centrifuged under the same conditions. There is a second wash step, after which the pelleted hemocytes are re-suspended in 500 µl of ice-cold IPS.

****Step 6: Quantification of hemocytes (Day 3)****. We mix 10 µl of suspended hemocytes with 10 µl of 0.4% trypan blue dye, then load 10 µl of the mixture beneath the cover slip of a hemocytometer cleaned with 70% ethanol. Live cells do not stain with trypan blue dye. The hemocytometer is observed under 10 × magnification on a microscope. Cell counts are made in the four outer squares, and the average of the four is calculated, then doubled to account for the dilution with trypan blue strain. This value is used to adjust the concentration of the hemocytes to 2 × 10 ^5^ cells/ml in the culture plate.

****Step 7: Seeding of hemocytes (Day 3)****. We use 24-well plastic cell culture plates. The required volume of hemocyte suspension is added to three wells in a row, one row per treatment. The suspension is made up to 1 ml using Grace’s Insect Medium supplemented with L-glutamine and sodium bicarbonate + 2% penicillin-streptomycin solution + 2.5 µg/ml amphotericin B + 10% heat-inactivated foetal bovine serum at room temperature. Separate plates are made up for different time points as testing is generally destructive. The plates are placed in a vented lidded box containing damp blue roll, and incubated in a non-CO _2_ incubator at the appropriate temperature – either 28°C (the usual temperature for the culture of insect cells) or 37°C (the mammalian body temperature).

****Step 8: Viability counts (Day 3)****. Once seeded, the T0 plate is used for viability counts. A total of 10 µl of suspension are withdrawn from each well and mixed with 10 µl of 0.4% trypan blue stain, then 10 µl of the mixture is loaded beneath the cover slip on a 70% ethanol-cleaned hemocytometer. Cells in the four outer corners are counted after trypan blue staining and approximately 95% of the cells should exclude trypan blue strain indicating they are viable.

****Step 9: Maintenance of hemocytes (Day 5–7)****. We check the hemocytes under 10 × magnification on the microscope to make sure that they are intact and attached to the bottom of the plate. We carry out a 50% media change using Grace’s Insect Medium supplemented with L-glutamine and sodium bicarbonate + 2% penicillin-streptomycin solution + 2.5 µg/ml amphotericin B + 10% heat-inactivated foetal bovine serum at room temperature as before.

****Step 10: Viability counts (Day 10)****. We pre-warm trypsin + 0.25% EDTA in a 37°C water bath. The plate is removed from the incubator and the medium withdrawn from each well. We use 1 ml of Dulbecco’s PBS to wash each well, × 2. We add 200 µl of the warmed trypsin + 0.25% EDTA to each well, and incubate the plate in an incubator at 37°C for 3 minutes. We inactivate the trypsin by addition of 400 µl of room temperature Grace’s Insect Medium supplemented with L-glutamine and sodium bicarbonate + 2% penicillin-streptomycin solution + 2.5 µg/ml amphotericin B + 10% heat-inactivated foetal bovine serum, pipetting the mixture slowly across the base of each well at least twice. The bases of the wells are also tapped sharply. The plate is observed at 10 × magnification under the microscope to ascertain whether the hemocytes are detaching from the surface of the well; if not, they are gently scraped with a 10 µl plastic loop. Once hemocytes are sufficiently detached, 10 µl from each well is mixed with 10 µl of trypan blue stain, and viability counts are undertaken as before. Typically, 94–97% of the cells are viable at this stage.

****Step 11: Differential hemocyte counts (Days 3 – 10)****. Seeded wells are examined under 20 × magnification on the microscope, and counts are made across five views of the three wells per treatment of granulocytes, spherulocytes, plasmatocytes, prohemocytes or oenocytoids according to their morphology. Giemsa staining facilitates the identification of the different types of hemocytes because the cell and nuclei shape are more easily observed.

****Step 12: Giemsa staining (Days 3 – 10)****. If we are undertaking Giemsa staining, we set up extra wells in the 24-well plate as this is a destructive process. The medium is removed and the wells are washed with 1 ml of room temperature Dulbecco’s PBS. Once this is removed, they are allowed to air dry briefly. We add 200 µl of undiluted Giemsa stain to each well and leave the plate for 2 minutes at room temperature. The stain is removed as thoroughly as possible. We add 200 µl of room temperature sterile deionized water and leave the plate for 3 minutes before removing as much water as possible. The wells are rinsed with a further 200 µl of deionized water, which is pipetted off as thoroughly as possible; the plate is then left to air dry before being examined at 20 × magnification on the microscope.

## Results

### Characterisation studies

**Figure 3.**
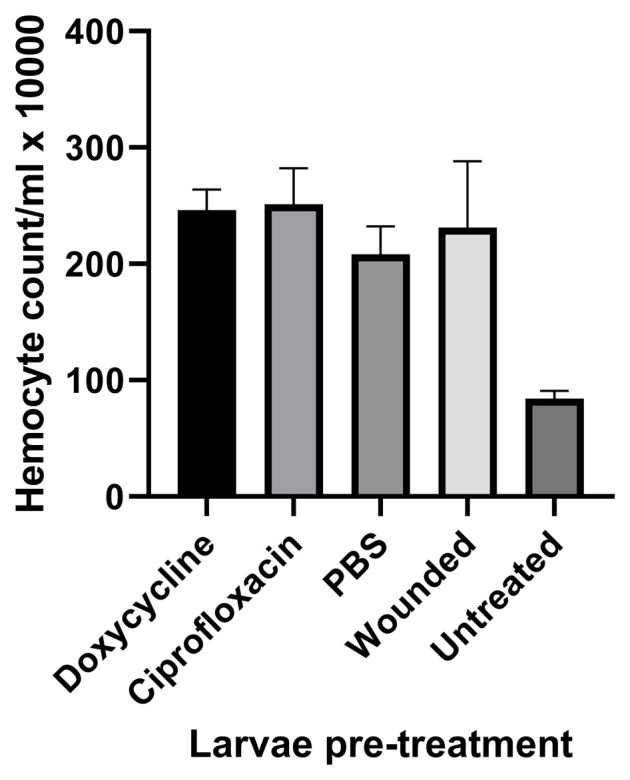
Hemocytes recovered from larvae pre-dosed with the antibiotic indicated, PBS or wounded with a syringe needle for three days prior to hemocyte recovery. The results shown are the average of four counts made on hemolymph extracted from groups of 10 larvae per treatment. Error bars = SD.

***Injection promotes the mobilisation of hemocytes.*** We initially investigated whether dosing larvae with an antibiotic affected the recovery of hemocytes compared to sham injected, PBS dosed or untreated control larvae. Pre-treating larvae with doxycycline, ciprofloxacin or PBS increased by 2–3-fold the number of hemocytes we recovered in hemolymph, compared to untreated controls ( Figure 3). However, sham injection by the insertion of a needle without any injection also increased the numbers of hemocytes recovered. The significance of these results could not be tested as hemolymph from each experimental unit was pooled into treatment sets and we thus do not have separate results for each larva.

**Figure 4.**
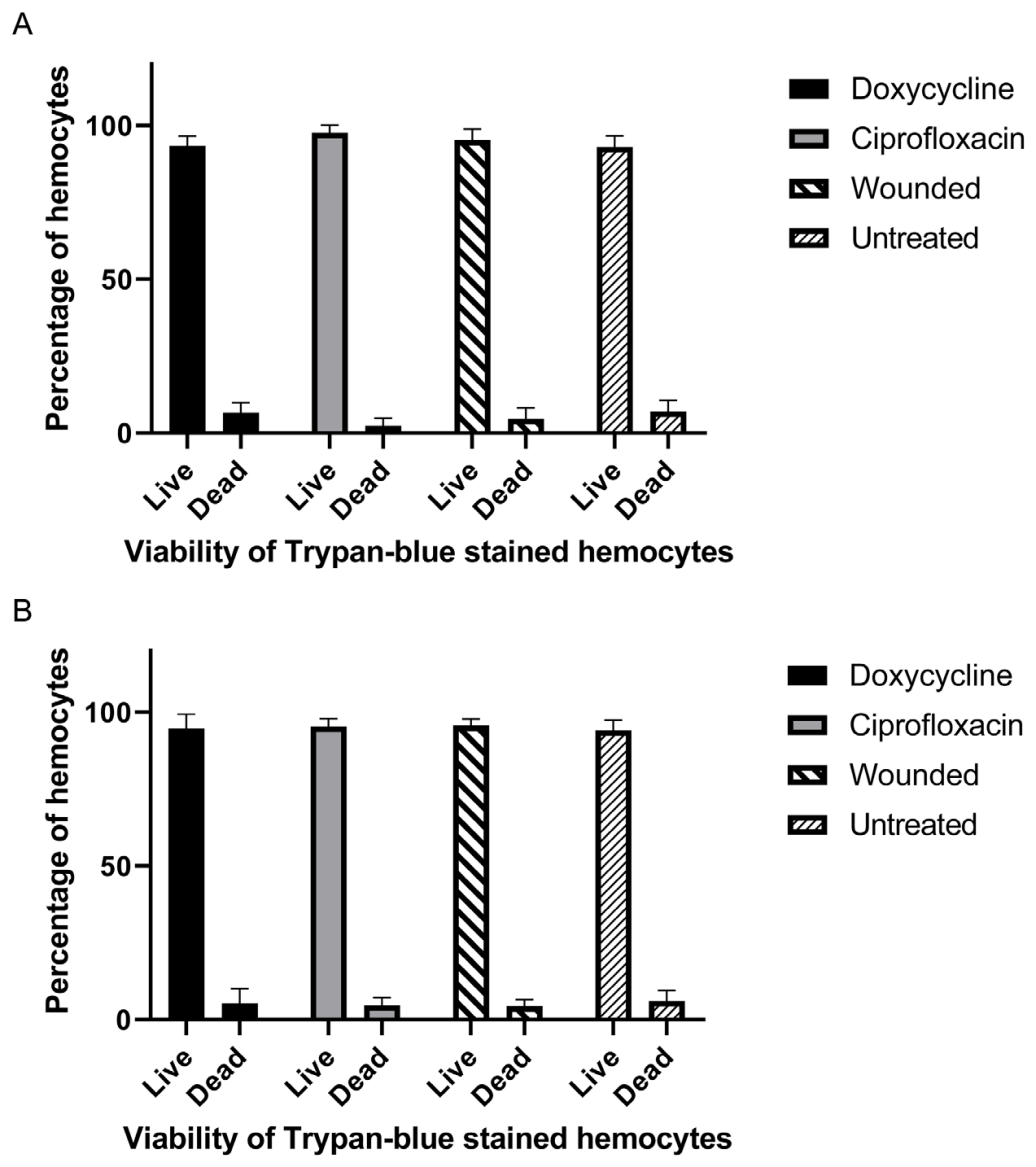
Viability of hemocytes incubated at 28ºC at T0 ( **A**) or T7 ( **B**). Average across three replicate wells per treatment, derived from the pooled hemolymph from 10 larvae, and seeded at 2 × 10 ^5^ cells/ml. Hemocytes dissociated with trypsin and stained with trypan blue. Error bars = SD. Mean percentage (and SD) of hemocytes live at T0: doxycycline treated, 93% (±3.2%); ciprofloxacin treated, 98% (±2.5%); wounded, 96% (±3.5%); untreated, 93% (±3.6%). Mean percentage (and SD) of hemocytes live at T7: doxycycline treated, 95% (±4.7%); ciprofloxacin treated, 95% (±2.5%); wounded, 96% (±2.1%); wounded, 94% (±3.5%).

***Survival of recovered hemocytes.*** We collected the hemocytes from hemolymph by centrifugation, and suspended them in Grace’s Insect Medium supplemented with L-glutamine, sodium bicarbonate, penicillin, streptomycin, amphotericin and FBS. The hemocytes were plated at a density of 2 × 10 ^5^ cells/ml into 24 well cell culture plates at 28°C. We found ( Figure 4) that the proportion of cells which excluded trypan blue dye (i.e. live cells) immediately after plating (93–98%) was similar to the proportion of cells which excluded trypan blue dye 7 days after plating (94–97% of T0 cells), indicating that we could maintain the cells for 7 days without loss of viability. Pre-treatment of the larvae with doxycycline or ciprofloxacin, before harvesting the hemocytes, did not affect the 7-day survival of hemocytes. When we cultured the hemocytes for 14 days after plating, we found a reduction in the proportion of cells which excluded trypan blue dye (33–38%).

**Figure 5.**
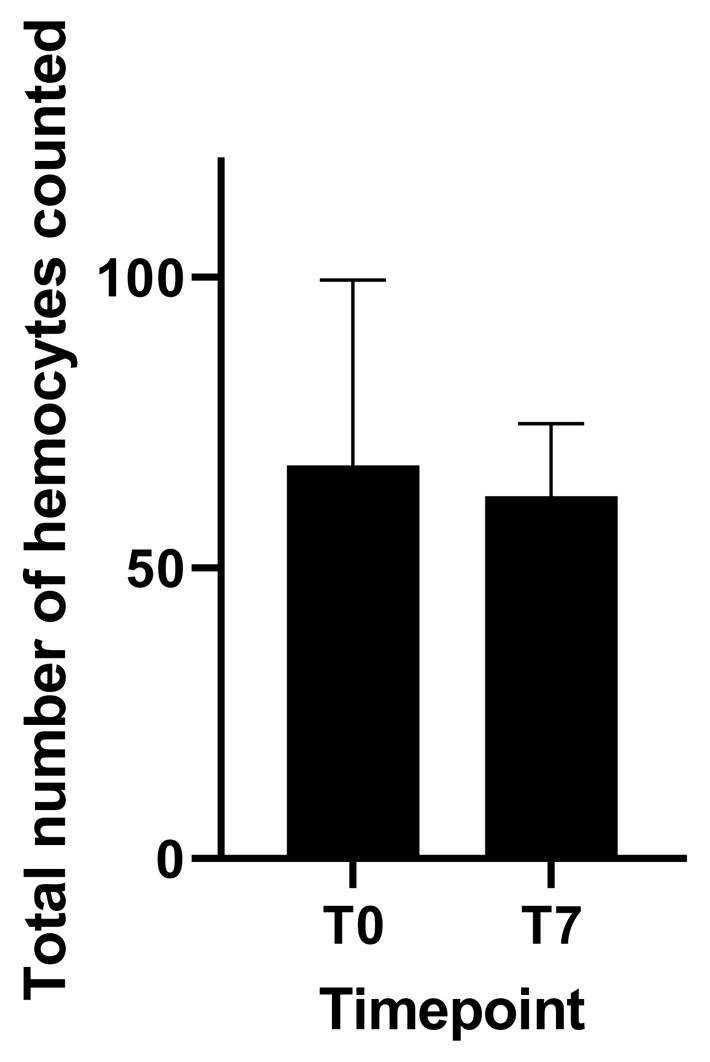
Total hemocyte count of cells in 10 µl from sham injected larvae incubated at 28°C at T0 and T7. Average across three replicate wells, derived from the pooled hemolymph from 10 larvae and seeded at 2 × 10 ^5^ cells/ml. Hemocytes stained with trypan blue and dissociated with trypsin at T7 only. Errors bars = SD. Mean (and SD) cell counts of hemocytes at T0; 68 (±32) or at T7; 62 (±12.5).

The total number of hemocytes, observed by microscopy, was similar at T0 and T7 ( Figure 5) with mean cell counts of 68 (standard deviation ±32) and 62 (standard deviation ±13), respectively. This suggests that we did not see replication of the hemocytes. However, it is also possible that there was balanced growth and division and corresponding death of hemocytes.

**Figure 6.**
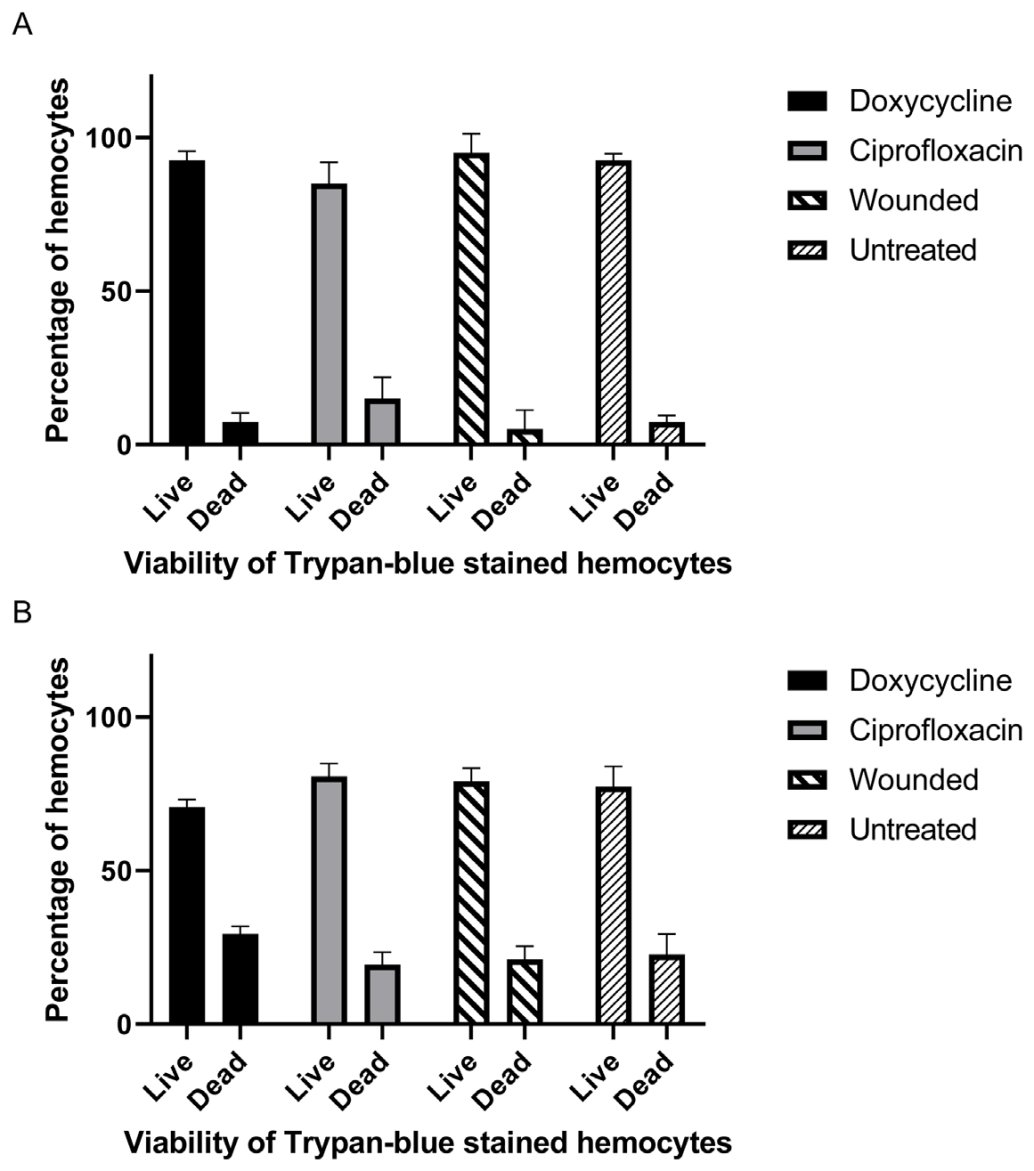
Viability of *ex vivo* hemocytes incubated at 37°C at T0 ( **A**) and T7 ( **B**). Average across 3 replicate wells per treatment, derived from the pooled hemolymph from 10 larvae and seeded at 2 × 10 ^5^ cells/ml. Hemocytes were dissociated with trypsin and stained with trypan blue. Error bars = SD. Mean percentage (and SD) of hemocytes live at T0: doxycycline treated 93% (±3); ciprofloxacin treated, 85% (±6.9); wounded, 95% (±6.2); untreated, 93% (±2.1). Mean percentage (and SD) of hemocytes live at T7: doxycycline treated 71% (±2.5); ciprofloxacin treated, 81% (±4.2); wounded, 79% (±4.4); untreated, 77% (±6.7).

***Hemocyte survival at 37°C.*** We next repeated the study outlined above, but incubated the hemocytes at 37°C ( Figure 6). We found that 71%–81% of the hemocytes excluded trypan blue day 7 days after isolation from larvae compared to 85%–95% at T0. The dosing of larvae with doxycycline or ciprofloxacin before harvesting the hemocytes did not affect their subsequent survival at T7, compared to the survival of hemocytes isolated from larvae dosed with PBS or sham injected. Overall, our results indicate reduced survival of hemocytes maintained at 37°C compared to 28°C. However, the numbers of cells that survive for 7 days at 37°C were sufficient to allow experimental studies during this time.

***Differential hemocyte counts.*** Unstained hemocytes isolated from larvae that were sham injected were assigned into different groups depending on their appearance by microscopy. We identified five forms of cells which we termed granulocyte-like, spherulocyte-like, plasmatocyte-like, prohemocyte-like or oenocytoid-like ( Table 3). After staining with Giemsa, we were able to visualise the differences between the hemocyte sub-populations as imaged in the report by Arteaga Blanco *et al* ^15^. The representation of the different hemocyte types was broadly similar at the time of isolation (T0) or after culture for 7 days (T7). However, there was a small reduction in the total number of hemocytes recorded ( Figure 6) and there was a reduction in the proportion of spherulocyte-like cells and an increase in the proportion of prohemocyte-like cells. Cell counts for all experiments, in addition to the raw image files used to generate figures, are available as *Underlying data* ^21^.

**Table 3.** Percentages of different hemocyte cell types at T0 or T7, isolated from sham injected larvae. Differential hemocyte counts were based on morphological differences of unstained cells under 20 × magnification. Percentages shown are the averages across five views per well from triplicate wells of hemolymph pooled from 10 larvae.

Timepoint	Granulocyte- like cells	Spherulocyte- like cells	Plasmatocyte- like cells	Prohemocyte- like cells	Oenocytoid- like cells
T0	56.5%	25.1%	5.3%	11%	2.1%
T7	56.7%	11.9%	2.5%	27%	1.7%

## Discussion

We report here a method for the reliable isolation of hemocytes for further studies on the biology of these cells. The method is easily transferred to other laboratories and requires no specialist techniques or equipment. Since the hemocytes survive for at least 7 days as primary cultures, even at 37°C, it should be possible to study the uptake, growth and survival of a range of pathogens within these phagocytes. This will allow the behaviour or pathogens in insect hemocytes to be compared with the behaviour of pathogens in mammalian phagocytes. In the longer term, an increased understanding of the behaviour of *G. mellonella* hemocytes will increase our understanding of the limitations of using insects to model infectious diseases of mammals.

Previously it has been reported that dosing with a sub-lethal dose of pathogen or a microbial component can evoke immune priming in *G. mellonella* larvae, where hemocytes which are normally bound to the inner surface of the cuticle become activated and mobilised into the hemolymph ^22^. A similar response can be elicited by some antimicrobials and by physical stresses such as physical agitation of the larvae or temperature changes ^22^. Our findings show that injury is another stress that can cause hemocyte mobilisation. Our findings highlight the importance of including a sham-injected control group in infection studies.

We did not see evidence of growth and replication of hemocytes in our study. It is likely that hemocytes require the presence of additional factors in order to replicate ^23^. Yamashita and Iwabuchi ^16^ reported that prohemocytes from *Bombyx mori* were more likely to divide if larval hemolymph was included in the culture medium. Further work should investigate how the replication of hemocytes can be encouraged *ex vivo*.

The hemocyte types that we found are similar to the types previously reported by Arteaga Blanco *et al*. ^15^ and Gwokyalya and Altuntaş ^24^ though other workers have reported different sub-populations of hemocytes ^25– 27^. Granulocytes are considered to be the most abundant type of hemocyte and one of the main types of phagocytic cell ^14^. They would be expected to be mobilised following injection or wounding. Plasmatocytes are the other main type of phagocyte ^14^. Whilst our findings broadly reflect those of Arteaga Blanco *et al*. ^15^ and Gwokyalya and Altuntaş ^24^, there are some differences in the proportions of different hemocyte types. These previous studies concluded that granulocytes and plasmatocytes together formed the majority of hemocytes (81.6–87.8% and 93.8–96%, respectively), whereas our predominant sub-populations at T0 were granulocyte-like and spherulocyte-like cells (81.6%). The method we have reported here will allow further work to investigate whether pathogens interact in different ways with the different sub-populations of hemocytes and to study whether some sub-populations are more able to eliminate pathogens than others. This information might provide new insight into the different roles of the hemocyte sub-populations. One important goal of any future research should therefore be to develop methods for the selective enrichment of the individual sub-populations of hemocytes.

In summary, we have optimised a protocol for the extraction of hemocytes from *G. mellonella* and the maintenance of these hemocytes *ex vivo* for a period of at least 7 days. The method we report here will now allow other investigators to isolate hemocytes and study the interactions of different types of hemocytes with pathogens. Our work also raises the possibility that protection against infection seen after the administration of drugs may be a consequence of the mobilisation of hemocytes as a consequence of the traumatic injury suffered.

## Data availability

### Underlying data

Open Science Framework: Isolation and primary culture of Galleria mellonella hemocytes for infection studies. https://doi.org/10.17605/OSF.IO/C97DT ^21^.

This project contains the following underlying data:

- Hemocyte_counts_treated_untreated_larvae_sets (numbers of hemocytes recovered from pooled hemolymph drawn from treated and untreated larvae sets)
- T0_hemocyte_viability_28C (numbers of live and dead hemocytes recovered from pooled hemolymph, incubated at 28°C and assessed at T0)
- T0_hemocyte_viability_37°C (numbers of live and dead hemocytes recovered from pooled hemolymph, incubated at 37°C andassessed at T0)
- T7_hemocyte_viability_28C (numbers of live and dead hemocytes recovered from pooled hemolymph, incubated at 28°C and assessed at T7)
- T7_hemocyte_viability_37°C (numbers of live and dead hemocytes recovered from pooled hemolymph, incubated at 37°C and assessed at T7)
- Total_hemocyte_count_wounded (total number of hemocytes in 10 µl of cell suspension from sham injected larvae, incubated at 28°C, assessed at T0 and T7)
- Figure_3_hemocyte_counts_treated_untreated (bar chart of numbers of hemocytes recovered from pooled hemolymph drawn from treated and untreated larvae sets)
- Figure_4A_T0_hemocyte_viability_28C (bar chart of percentage of live and dead hemocytes recovered from pooled hemolymph, incubated at 28°C and assessed at T0)
- Figure_4B_T7_hemocytes_viability_28C (bar chart of percentage of live and dead hemocytes recovered from pooled hemolymph, incubated at 28°C and assessed at T7)
- Figure_5_total_hemocyte_count_wounded (bar chart of total number of hemocytes in 10 µl of cell suspension from sham injected larvae, incubated at 28°C, assessed at T0 and T7)
- Figure_6A_T0_hemocyte_viability_37C (bar chart of percentage of live and dead hemocytes recovered from pooled hemolymph, incubated at 37°C and assessed at T0)
- Figure_6B_T7_hemocyte_viability_37C (bar chart of percentage of live and dead hemocytes recovered from pooled hemolymph, incubated at 37°C and assessed at T7)

Data are available under the terms of the Creative Commons Zero "No rights reserved" data waiver (CC0 1.0 Public domain dedication).

## Author roles

**Titball R**: Conceptualization, Data Curation, Formal Analysis, Investigation, Methodology, Project Administration, Resources, Validation, Visualization, Writing – Review & Editing; **Senior, N**: Experimental work, Data Curation, Formal Analysis, Investigation, Methodology, Writing – Original Draft Preparation, Writing – Review & Editing
