# Supplementary material for: Isolation and primary culture of Galleria mellonella hemocytes for infection studies
Source: F1000Res. 2021 Feb 16;9:1392. Originally published 2020 Dec 3. [Version 2] doi: 10.12688/f1000research.27504.2 (PMC7818094; doi:10.12688/f1000research.27504.2)
Supplement: Peer review history

# Peer review: Reviewer response for version 1

**DOI:** 10.5256/f1000research.30397.r77334

**Published:** 2021-01-20

**Authors:** Daniel R. Neill, Joanne L. Fothergill

**Affiliations:** 1 Institute of Infection, Veterinary and Ecological Sciences, University of Liverpool, Liverpool, UK

The manuscript by Senior and Titball describes a novel method for isolation and culture of hemocytes from *Galleria mellonella* larvae. These **larvae are, as the authors point out, increasingly used as a cheap, reliable and more ethically acceptable alternative to mammalian species, for the study of infection and in therapeutic development. Despite this, methodologies for working with *G. mellonella *are not standardised and many aspects of the larvae's biology are not well described. This paper sets out to address some of these knowledge gaps, and makes several important contributions to the field.

 Firstly, a method for reliable isolation of hemocytes is described. Sufficient detail is given for others to be able to adopt the method and we expect that it will be widely used. The authors highlight a number of potential applications in the fields of immunology, infection and therapeutics and there is clear potential for exploitation of the model in all these areas.

 Secondly, the authors describe the effect of needle injury on hemocyte recovery and highlight the implications this has for experiments performed with *G. mellonella*. We agree with the authors that the increased recovery of immune cells following needle injury (in the absence of substance administration) demonstrates the importance of including sham injected controls in experiments.

 The manuscript is clear and well-written throughout and represents an important step forward for the field, with obvious 3Rs implications. We have a few minor comments that might help readers better understand and utilise the model:

1. One use of this model would be to look at hemocyte responses in infected larvae. Such experiments would likely be performed at 37C. The authors provide nice data on hemocyte survival *ex vivo *at 37C but can they comment on how recovery differs when the larvae are maintained at 37C, rather than 15C, prior to harvesting the hemocytes?
2. To the uninitiated, the terms used for the hemocyte sub-types will likely mean little. Microscopy images would be useful to enable users to distinguish between subtypes in their cultures.
3. The authors state that using pooled hemocyte cultures prevents statistical analysis when the individual larvae is treated as the experimental unit, which is fine, but this doesn't seem practical for most users, who will want to statistically compare cultures. Can the authors comment on sample size determination for future studies that treat individual culture wells as the experimental unit? The mean and S.D. of recovery and viability per larvae is given, but are there other factors that need to be considered, such as the rate of bacterial contamination of recovered cells? 

Is the rationale for developing the new method (or application) clearly explained?

Yes

Are the 3Rs implications of the work described accurately?

Yes

Is the description of the method technically sound?

Yes

If applicable, is the statistical analysis and its interpretation appropriate?

Yes

Are the conclusions about the method and its performance adequately supported by the findings presented in the article?

Yes

If any results are presented, are all the source data underlying the results available to ensure full reproducibility?

Partly

Are a suitable application and appropriate end-users identified?

Yes

Are sufficient details provided to allow replication of the method development and its use by others?

Yes

Reviewer Expertise:

Infection models and host-pathogen interactions. Referee suggested by the NC3Rs for their scientific expertise and experience in assessing 3Rs impact.

We confirm that we have read this submission and believe that we have an appropriate level of expertise to confirm that it is of an acceptable scientific standard.

# Response

**Published:** 2021-02-10

**Authors:** Nicola Senior

**Affiliations:** University of Exeter, UK

We thank the reviewers for the time they have invested in improving our manuscript.

 Response to point 1. This is an interesting point raised by the reviewers. For infection studies using *G. mellonella* larvae we normally maintain them at 15C, and then transfer them to a higher temperature after challenge. This is a protocol used by most other researchers. We have used this protocol in the studies reported here, where hemocytes are harvested from larvae at 15C, but then incubated at 37C (or 28C where indicated). We do not have any data on the yield or properties of hemocytes taken from larvae which had been held at 37C before harvesting the cells. This is certainly a study that other investigators might want to undertake.

 Response to point 2. We agree that images would be useful. We do not have publication quality images to hand but will now aim to collect these images.

 Response to point 3. It would be feasible to include 3 or 4 groups of 10 larvae in each experimental unit, allowing statistical analysis to be undertaken. We did not observe any bacterial contamination in the studies we undertook, over the time period we reported, and we would not expect this to be a problem.

# Peer review: Reviewer response for version 1

**DOI:** 10.5256/f1000research.30397.r75815

**Published:** 2020-12-18

**Authors:** Lauren Ames

**Affiliations:** 1 Pacific Northwest Research Institute, Seattle, USA

This research paper details a novel method for the extraction and culture of hemocytes from the wax moth larva, *Galleria mellonella*. This invertebrate host has become routinely used for studying the virulence of microbial pathogens and antimicrobial drug efficacy. However, current *G. mellonella *infection models are mostly limited to measuring larval survival as the main data output. In this study, the authors found that hemocytes can be extracted from *G. mellonella *larvae and maintained *ex vivo *for seven days post isolation with high viability. These findings are important because they broaden the *G. mellonella* infection model toolbox to include the study of pathogen-immune cell interactions. The authors also note increased hemocyte recovery from needle stick injured larvae, highlighting the importance of including relevant controls in routine *G. mellonella *survival experiments.

 The method in this paper is described with high attention to detail and the results are clearly presented. Conclusions drawn are strongly supported by data from carefully thought-out experiments. The work contributes to the development of invertebrate infection models which is vital for reducing the use of mammalian hosts. My only suggestion is that a figure showing representative images of the hemocyte subpopulations identified could be useful to a researcher using this method for the first time.

Is the rationale for developing the new method (or application) clearly explained?

Yes

Are the 3Rs implications of the work described accurately?

Yes

Is the description of the method technically sound?

Yes

If applicable, is the statistical analysis and its interpretation appropriate?

Yes

Are the conclusions about the method and its performance adequately supported by the findings presented in the article?

Yes

If any results are presented, are all the source data underlying the results available to ensure full reproducibility?

Yes

Are a suitable application and appropriate end-users identified?

Yes

Are sufficient details provided to allow replication of the method development and its use by others?

Yes

Reviewer Expertise:

Medical mycology, invertebrate models of fungal infection, yeast genetics, antifungal drugs.

I confirm that I have read this submission and believe that I have an appropriate level of expertise to confirm that it is of an acceptable scientific standard.

# Response

**Published:** 2021-02-10

**Authors:** Nicola Senior

**Affiliations:** University of Exeter, UK

We thank the reviewer for the time they have invested in improving our manuscript.  We agree that images would be useful.  We do not have publication quality images to hand but will now aim to collect these images.
